# Parent’s food literacy and adolescents nutrition literacy influence household’s food security and adolescent’s malnutrition and anemia: Findings from a national representative cross sectional study

**DOI:** 10.3389/fnut.2022.1053552

**Published:** 2022-12-30

**Authors:** Maha Hoteit, Hala Mohsen, Lara Hanna-Wakim, Yonna Sacre

**Affiliations:** ^1^Faculty of Public Health, Lebanese University, Beirut, Lebanon; ^2^Faculty of Public Health, PHENOL Research Group (Public HEalth Nutrition prOgram Lebanon), Lebanese University, Beirut, Lebanon; ^3^Lebanese University Nutrition Surveillance Center (LUNSC), Lebanese Food Drugs and Chemical Administrations, Lebanese University, Beirut, Lebanon; ^4^University Medical Center, Lebanese University, Beirut, Lebanon; ^5^Department of Agricultural and Food Engineering, School of Engineering, Holy Spirit University of Kaslik (USEK), Jounieh, Lebanon; ^6^Department of Nutrition and Food Sciences, Faculty of Arts and Sciences, Holy Spirit University of Kaslik (USEK), Jounieh, Lebanon

**Keywords:** food literacy, nutrition literacy, food insecurity, malnutrition, anemia, parents-adolescents dyads

## Abstract

**Introduction:**

Food and nutrition literacy are widely fundamental to improve the food security and reduce the double burden of malnutrition and iron deficiency in low- and middle-income countries.

**Objectives:**

This study aimed (1) to assess the nutrition literacy (NL) of Lebanese adolescents and their parents’ food literacy (FL), (2) to investigate the impacts of adolescents’ NL and parental FL on (a) the household food security, (b) the adolescents’ self-reported food security, and (c) the adolescents’ nutrition status with a focus on malnutrition and anemia.

**Methods:**

A national representative sample of 450 parent–adolescent dyads [parents: mean age ± standard deviation (SD) = 46.0 ± 7.0, mothers: 59.0%; adolescents: mean age ± SD = 15.0 ± 3.0, girls: 54.6%] were interviewed. Anthropometric and blood hemoglobin measurements were performed for adolescents. The Arab Family Food Security Scale and the Adolescent-Level Food Security Scale were used.

**Results:**

Around 45.0% of the adolescents were nutritionally illiterate, and nearly half (47.8%) of parents had poor FL. Around 68.2 and 54.0% of the households and adolescents were food insecure, respectively. Moreover, 6.7, 4.7, 32.2, and 16.7% of the adolescents were stunted, thin, overweight/obese, and anemic, respectively. Poor parental FL increased the risk of household food insecurity (FI) by 2.7 times, *p* < 0.001. Adolescents’ nutrition illiteracy increased their FI risk by 60.0% (*p* = 0.02). The number of offspring, household income, crowding status, and participants’ residence also influenced the percentage of FI and malnutrition prevalence among households and adolescents.

**Conclusion:**

Improvements in FL and NL are promising to mitigate FI malnutrition in Lebanon.

## Introduction

Food and nutrition literacy are nowadays considered as the bridge between food, nutrition, and well-being of communities in low-and middle-income countries (1). Moreover, it can serve as a fundamental step toward the capacity building to effectively use nutrition/food knowledge and skills, to meet specifically the adolescent’s current and future health (1). Many definitions and conceptualizations of food/nutrition literacy are available; however, a widely-cited definition describes food literacy as a “collection of interrelated knowledge, skills, and behaviours required to plan, manage, select, prepare, and eat foods to meet needs and determine food intake.” Furthermore, food literacy is “the staging that empowers individuals, households, communities, and nations to protect diet quality through change and support dietary resilience over time” (2). Some studies have characterized food literacy as the ability to search and understand nutrition-related information (3). (On the other hand, Lee, C.-K., and colleagues (4) defines nutrition literacy (NL) as a set of individual and context-related characteristics which allow adherence to a healthy diet that respects the guidelines on proper nutrition and recommendations. It concerns also the dietary performance, which reflects the competence of healthy eating patterns. A connection between the two concepts of FL and NL has been well documented frequently where both highlighted that food behaviors, skills, and knowledge cannot be separated because they are part of the same construct that is FNL (1–4). Exploring the status of NL in the early years of life, particularly during adolescence, is crucial and may aid in the adaptation of sustainable nutrition interventions (5). Adolescents are more prone to dietary vulnerabilities because they are going through a nutrition-sensitive phase of rapid growth and development (6). According to many solid evidences, NL has appeared as a critical factor in promoting and maintaining healthy dietary practices among adolescents, including food label use (7), higher dietary diversity (8), and nutrient intake adequacy (8). In addition, a higher prevalence of overweight and obesity was observed among nutritionally-illiterate adolescents (9). Not only improving nutrition outcomes but advocating for better FL and NL could help build resilience against food insecurity (FI) (10). FI is the state of being unable to access a sufficient quantity and quality of food (11). The four pillars of food security are (1) food availability; (2) food access; (3) food utilization; (4) stability of these three pillars over time (11). Of them, the utilization pillar encompasses FL skills and knowledge, which determines how the individual store, prepare and cook food (12). Food illiteracy may impede the adequate utilization of the available food and worsens the food security status even for wealthy communities (12). Begley et al. showed that households experiencing FI typically have worse levels of FL, manifested by low cooking self-efficacy, and unfavorable food purchasing habits (10). As well, Khorramrouz accentuates that there is a negative relationship between children’s NL and their food security status (13). These lessons are to be considered in Lebanon, where FI has become inescapable for almost all Lebanese households (14–16). The Lebanese people are struggling to recover from the economic distress they face daily in the marketplace and when gathering with their families for meals.. Based on the above, this study aims to: (1) to assess the nutrition literacy (NL) of Lebanese adolescents and their parents’ food literacy (FL), (2) to investigate the impacts of adolescents’ NL and parental FL on (a) the household food security, (b) the adolescents’ self-reported food security (c) and the adolescents’ nutrition status with focus on malnutrition and anemia.

## Materials and methods

### Study design and participants’ recruitment

This was a cross-sectional study including a representative sample of Lebanese parent-adolescent dyad, conducted from mid-March to mid-July 2022. Households were recruited using the stratified cluster sampling. The clusters were the eight Lebanese governorates (Mount Lebanon, Beirut, South Lebanon, North Lebanon, Akkar, Beqaa, Baalbeck-Hermel, and Nabatieh). Within each governorate, households were recruited using a probability proportional to size sampling technique. A single population formula was used to determine the sample size *n* = [*p* (1 – *p*)] * [(*Z*_∝/2_)^2^/(*e*)^2^], where *n* is the sample size, *Z* (∝/2) is the reliability coefficient of standard error at a 5% level of significance = 1.96, *p* = 0.05, and *e* refers to the level of standard error tolerated (5%) (17). Thus, it was determined that 400 parent-adolescent dyads are an adequate sample size to ensure sufficient power for statistical analyses. After accounting for a 10% non-response rate, 450 parent-adolescent dyads were included in our final sample. This sample size is sufficient to ensure appropriate power for statistical analyses based on the statistics of the central Administration of Statistics in Lebanon (18).

### Inclusion and exclusion criteria

**FIGURE 1:**
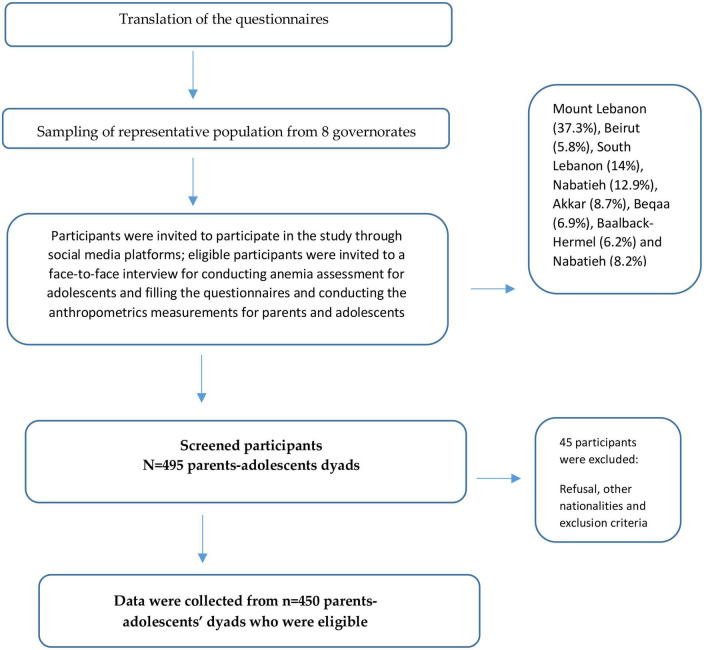
Flow diagram of the recruitment process in the current study.

A total number of 495 healthy adolescents, from both genders and aged 10–19 years old were recruited. Adolescents who were using iron dietary supplements, and donated blood in the month preceding the assessment were excluded from the study. In addition, only one adolescent child from each household was enrolled in the study. Parents with Lebanese nationality only and aged 18–64 years old were recruited (Refer to Figure 1).

### Instruments and measures

Using a validated and well-designed questionnaire (19), the following information were collected from participants: (i) parents: age, gender, residency, marital status, the number of children the parent has, education level, occupation status, family monthly income (and if they experienced a recent decline in the income), the number of co-residents in the home, and the number of rooms; (ii) adolescents: date of birth, gender, residency, primary caregiver, whether currently working, education level, school type, and whether the adolescent receives nutrition education in their schools.

### Adolescents’ nutrition literacy

The adolescent’s NL status was assessed using a valid 22-items scale: The Adolescent Nutrition Literacy Scale (ANLS) (20). This questionnaire was translated from a Turkish study to Arabic language. It was revised and translated by experts. ANLS evaluated three components of NL: Functional NL (FNL), Interactive NL (INL), and Critical NL (CNL). FNL reflects the capacity to comprehend nutrition concepts (21). INL refers to the communication skills required to receive nutrition information (21). CNL reflects the ability to critically analyze, evaluate and apply nutrition information (21). Each question in the ANLS has a score ranging from 0 to 5 (5-points Likert scale: strongly disagree to strongly agree). The questionnaire has an overall score range from 22 to 110 (a higher score indicates better NL). Adolescents’ NL was considered poor or adequate based on the median scores (TNL: 68.0; FNL = 21.0; INL = 18.0; CNL = 30.0).

### Parent’s food literacy

A valid 12-item “short food literacy questionnaire ‘SFLQ’ (21) was used to evaluate parent’s FL”. This questionnaire was translated from a Swiss study to Arabic language. It was revised and translated by experts. Respondents answered via four- or five-point Likert scales. The questionnaire had a score ranging from 7 to 52. A higher score suggests better food literacy. The median score of 31.0 was used to categorize parents into having poor food literacy or adequate food literacy (22).

### Household and adolescents’ self-reported food security

The Arab Family Food Security Scale (AFFSS) (23), and a self-reported adolescent-level-food security scale (24) were used. These valid scales classified adolescents and their households as being either “food secure” or “foodinsecure.”

### Parents and adolescents’ nutrition status

Parents and adolescents’ body weight was measured to the nearest 0.1 kg while ensuring light clothing with no shoes using an electronic scale (Amber Body scale, NUMED SARL, Beirut, Lebanon). The height was measured to the nearest 0.1 kg barefooted using a portable stadiometer (Portable Height scale; NUMED SARL, Beirut, Lebanon). The adolescent’s body weight and height were then entered into the 2009 WHO AnthroPlus software and expressed as Z-scores [Height-for-age (HAZ) and BMI-for-age (BAZ)]. Afterward, the adolescent nutrition status was classified as the following: stunting: HAZ ≤ 2 SD; thinness: BAZ ≤ 2 SD; overweight: BAZ > 1 SD; obesity: BAZ > 2 SD (25). As for the parents, the body mass index (BMI) was assessed: values below 18.5 indicates underweight; 18.5–24.9: healthy weight; 25.0–29.9: overweight; 30.0 and Above: Obesity.

For anemia assessment, a portable POC hemoglobin analyzer (CompoLab TS) was used. This is non-invasive anemia detection technology that collect blood through finger pricks method. Following the WHO criteria (26), anemia was diagnosed as the following: boys and girls (younger than 12 years old): Hgb < 11.5 g/dl; boys and girls (12–14 years old): Hgb < 12 g/dl; boys (aged 15 years and older: Hgb < 13 g/dl; girls (aged 15 years and older: Hgb < 12.0 g/dl).

A flow diagram describing the recruitment process is shown in Figure 1.

### Ethical approval

This study’s ethical approval was granted by the Al-Zahraa University Medical Center’s ethics committee in Beirut, Lebanon (Reference Nb 12-2022). Informed consent was obtained from all participants, verbally and written. The participants were aware of having the right to deny being part of this study and withdraw for any reason. We adhered to the Helsinki declaration guidelines while conducting this study.

### Data analysis

The data was exported to the Statistical Package of Social Sciences Software (SPSS) (Version 25.0. IBM Corp: Armonk, NY, USA) for analysis. Descriptive measures, including frequency (*N*), percentage (%), mean, and standard deviation (SD) were obtained to summarize our findings. The Mann–Whitney *U* test was used to detect mean differences of non-normal data. The chi-squared (χ^2^) test was used to determine associations between the study variables. The Fisher’s exact test was used when one or more of the cell counts in a 2 × 2 table are less than 5. The binary logistic regression (Backward stepwise LR method) was performed to determine the extent of contribution of parental FL and adolescents’ NL in determining the household and adolescents’ self-reported food security and adolescents’ nutrition status, along with the other factors. A *p*-value of 0.05 and below was considered significant for all analytical tests.

## Results

### Household food security status, demographic and socioeconomic characteristics of parents

Parents, as well as their adolescent children, were recruited representatively from all Lebanese governorates, predominately from Mount Lebanon (37.3%). Among the 450 sampled parents, 59.0% were females. Almost half of them were aged between 30 and 45 years old, and 49.0% were aged between 46 and 64 years old. Almost 48.4% of the parents had normal body weight, and 49.7% were overweight or obese. The majority of parents were married (95.8%), and 54.0% had 2–3 children. Most of the parents had a school education level (65.0%), and around half (49.3%) reported having no occupation. Around 23.0% of parents reported having no income or < 1.5 million Lebanese Pound (LBP) equivalent to 42$ (for a rate of 35,000 LBP for 1$) as family monthly income, with 21.8% reporting a recent decline in the income. Nearly half (45.0%) of parents were living in crowded homes. In addition, 68.2% of the households were food insecure (Table 1).

**TABLE 1:** Demographic and socioeconomic characteristics of parent participants and the household food security status.

	Overall (*N* = 450)		Females (*n* = 266)		Males (*n* = 184)		
	Mean	SD	Mean	SD	Mean	SD	*P*-value
Age in years	46.0	7.0	47.0	7.0	46.0	7.0	<0.001**
	*N*	%	*N*	%	*N*	%	
Adulthood stage							<0.001**
Early middle adulthood (30–45 years old)	230	51.0	158	59.4	72	39.1	
Late middle adulthood (46–64 years old)	220	49.0	108	40.6	112	60.9	
Area of residency							<0.001**
Mount Lebanon	168	37.3	80	32.5	88	43.1	
Beirut	26	5.8	19	7.7	7	3.4	
South Lebanon	63	14.0	46	18.7	17	8.3	
North Lebanon	58	12.9	36	14.6	22	10.8	
Akkar	39	8.7	24	9.8	15	7.4	
Beqaa	31	6.9	18	7.3	13	6.4	
Baalbeck-Hermel	28	6.2	10	4.1	18	8.8	
Nabatieh	37	8.2	13	5.3	24	11.8	
Weight status							0.11
Underweight	8	1.8	7	2.6	1	0.5	
Normal weight	218	48.4	128	48.1	90	48.9	
Overweight	146	32.4	79	29.7	67	36.4	
Obese	78	17.3	52	19.5	26	14.1	
Marital status							0.76
Married	431	95.8	254	95.5	177	96.2	
Divorced	9	2.0	5	1.9	4	2.2	
Widowed	10	2.2	7	2.6	3	1.6	
Number of children the parent has							0.42
One child	34	7.6	17	6.4	17	9.2	
2–3 children	243	54.0	142	53.4	101	54.9	
More than 3 children	173	38.4	107	40.2	66	35.9	
Highest education level							0.001*
No formal education	35	7.8	11	4.1	24	13.0	
School education level	292	64.9	174	65.4	118	64.1	
University education level	123	27.3	81	30.5	42	22.8	
Having current occupation							<0.001**
Yes	228	50.7	71	26.7	157	85.3	
No	222	49.3	195	73.3	27	14.7	
Monthly income of the family							0.13
None	35	7.8	25	9.4	10	5.4	
Less than 1.5 million LBP	67	14.9	33	12.4	34	18.5	
≥1.5 million LBP	294	65.3	171	64.3	123	66.8	
≤300 USD	36	8.0	24	9.0	12	6.5	
More than 300 USD	18	4.0	13	4.9	5	2.7	
The household has experienced a recent decline in the monthly income							0.34
No	352	78.2	204	58.0	148	42.0	
Yes	98	21.8	62	63.3	36	36.7	
**Household crowding status^(a)^**							
Not crowded	248	55.1	159	59.8	89	48.4	0.04*
Crowded	202	44.9	107	40.2	95	51.6	
Household food security status							0.35
Food-secure	143	31.8	80	30.1	63	34.2	
Food-insecure	308	68.2	186	69.9	121	65.8	

^(a)^Determined based on the calculated crowding index (CI) (CI = number of persons in home/number of rooms excluding the kitchen and bathroom). CI ≤ 1 (no crowding); CI > 1 (crowding); *significant at *p*-value < 0.05 for the χ^2^ test; **significant at *p*-value < 0.001 for the χ^2^ test.

### Adolescents’ demographic characteristics, nutrition status, and self-reported food security status

Among the adolescent sample, 54.6% were girls. Around 40.0% of the adolescents aged 10–13 years old, 27.1% were aged between 14 and 16 years old, and 32.9% were 17–19 years old. More girls (42.6%) than boys (21.1%) were in the late adolescence stage, *p* < 0.001. Almost all (93.6%) were living with both parents. A few (6.9%) of the adolescents were working. Most of the adolescents (79.6%) were school students (45.1% of them were at public schools), while the remaining (20.4%) had a university education level. Almost all the adolescents (91.0%) were not receiving nutrition education in the schools’ curriculum. Additionally, 6.7% of adolescents were stunted, 4.7% were wasted, 32.2% were overweight or obese, and 16.7% had anemia. Furthemore, 54.0% of the adolescents were food insecure (Table 2).

**TABLE 2:** Adolescents’ demographic characteristics and nutrition status, and the self-reported adolescent food security status.

	Overall (*N* = 450)		Girls (*n* = 246)		Boys (*n* = 204)		
	Mean	SD	Mean	SD	Mean	SD	*P*-value
Age (in years)	15.0	3.0	15.0	3.0	14.0	3.0	<0.001**
	*N*	%	*N*	%	*N*	%	
Adolescence stage							< 0.001**
Early adolescence (10–13 years old)	180	40.0	86	35.0	94	46.1	
Middle adolescence (14–16 years old)	122	27.1	55	22.4	67	32.8	
Late adolescence (17–19 years old)	148	32.9	105	42.6	43	21.1	
Primary caregiver							0.05*
Either parent	17	3.8	11	4.4	6	3	
Both parents	421	93.6	224	91.1	197	96.6	
Others	12	2.7	11	4.5	1	0.5	
Currently working							0.27
No	419	93.1	232	94.3	187	91.7	
Yes	31	6.9	14	5.7	17	8.3	
Education							< 0.001**
School education level	358	79.6	177	72.0	181	88.7	
University education level	92	20.4	69	28.0	23	11.3	
School type							0.09
Public	183	45.1	87	41.0	96	49.5	
Private	223	54.9	125	59.0	98	50.5	
Receiving nutrition education at school							0.80
No	325	90.8	160	90.4	165	91.2	
Yes	33	9.2	17	9.6	16	8.8	
Nutrition status							
Stunting	30	6.7	18	7.3	12	5.9	0.54
Thinness	21	4.7	5	2.0	16	7.8	0.004*
Overweight/obesity	145	32.2	71	28.9	74	36.3	0.09
Anemia	75	16.7	40	16.3	35	17.2	0.79
Adolescents’ self-reported food security status							0.59
Food-secure	207	46.0	116	47.2	91	44.6	
Food-insecure	243	54.0	130	52.8	113	55.4	

*Significant at *p*-value < 0.05 for the χ^2^ test; **significant at *p*-value < 0.001 for the χ^2^ test.

### Adolescents’ NL and Parents’s FL

Overall, around 45.0% of adolescents were nutritionally illiterate. In particular, 46.0% had poor FNL, 49.3% had poor INL, and 38.4% had poor CNL. More boys (52.5%) than girls were nutritionally illiterate (38.2%), *p* = 0.002. Boys’ FNL and INL were significantly worse than that of girls (52.5% of boys vs. 40.7% of girls had poor FNL; 54.4% of boys vs. 45.1% of girls had poor INL), *p* = 0.01. Almost 47.8% of the parents were food illiterate, particularly fathers (49.0% vs. mothers: 51.0%, *p* = 0.005; Table 3).

**TABLE 3:** The status of adolescents’ NL and parenteral FL.

Adolescents	Overall (*N* = 450)	Gender		*P*-value
		Girls (*n* = 246)	Boys (*n* = 204)	
	*N* (%)	*n* (%)	*n* (%)	
**TNL^(1)^**				
Poor	201 (44.7)	94 (38.2)	107 (52.5)	0.002*
Adequate	249 (55.3)	152 (61.8)	97 (47.5)	
**FNL^(2)^**				
Poor	207 (46.0)	100 (40.7)	107 (52.5)	0.012*
Adequate	243 (54.0)	146 (59.3)	97 (47.5)	
**INL^(3)^**				
Poor	222 (49.3)	111 (45.1)	111 (54.4)	0.05*
Adequate	228 (50.7)	135 (54.9)	93 (45.6)	
**CNL^(4)^**				
Poor	173 (38.4)	89 (36.2)	84 (41.2)	
Adequate	277 (61.6)	157 (63.8)	120 (58.8)	
Parents	Overall (*N* = 450)	Females (*n* = 266)	Males (*n* = 184)	*p*-value
FL^(5)^	*N* (%)	*n* (%)	*n* (%)	0.63
Poor	215 (47.8)	115 (46.7)	100 (49.0)	
Adequate	235 (52.2)	131 (53.3)	104 (51.0)	

^(1)^TNL, total nutrition literacy; ^(2)^FNL, functional nutrition litarcy; ^(3)^INL, interactive nutrition literacy; ^(4)^CNL, critical nutition literacy; ^(5)^FL, food literacy; *significant at *p*-value < 0.05.

Impact of adolescents’ NL and parental FL on the household’s and adolescents’ food security, the adolescents’ nutrition status with focus on malnutrition and anemia. Supplementary Table 1 shows that households residing in Baalbeck-Hermel had the highest percentage of FI (82.2%), *p* < 0.001. Besides, households composed of more than 3 children were the most food-insecure (76.9%), *p* < 0.001. FI predominated among households with uneducated parents (88.6%), *p* = 0.005 andwas most prevalent in households earning less than 1.5 million LBP (78.4%), *p* = 0.01. Crowded households had a higher percentage of FI (73.3%) than non-crowded ones (64.1%), *p* = 0.04. Around 78.1% of food illiterate parents were belonging to food-insecure households, *p* < 0.001 (Supplementary Table 1).

Adolescents residing in Akkar were most food-insecure (84.6%), *p* < 0.001. Adolescents experienced more FI if they were belonging to households earning less than 1.5 million LBP (62.7%) monthly income, *p* = 0.04. Besides, 60% of adolescents living in crowded households were food-insecure, *p* = 0.01. More than half of nutritionally illiterate adolescents (60.7%) were observed to have FI, *p* = 0.01.

As for stunting, thinness, overweight/obesity, and anemia, these were not influenced by adolescents’ NL or parental FL; however, they were associated with other study variables. For instance, adolescents studying at school were more stunted (8.1%) than those who were university students (1.1%), *p* = 0.02. Thinness was more prevalent among boys (7.8%) than girls (2.0%), *p* = 0.004. Households reporting a recent decline in their salary had the highest proportion of thin adolescent children (9.2%), *p* = 0.02. Additionally, 40.0% of overweight or obese parents had an overweight/obese adolescent child, *p* = 0.002. Lower parental education level (school vs. university level) was associated with a higher percentage of adolescent with overweight/obesity (36.6% vs. 26.8%), *p* = 0.009. Anemia was mostly prevalent among adolescents residing in Akkar (36.0), *p* = 0.01 (Supplementary Table 1).

### Bivariate analysis on the impact of NFL on parents-adolescents dyads’ nutrition status and food security

Depending on the bivariate analysis shown above, we determined the contribution of the adolescents’ NL and parental FL to predicting the household and adolescents’ self-reported FI. Table 4 shows that the percentage of household FI increased 2.7 times when parents had poor FL (OR = 2.7, CI = 1.8–4.3 *p* < 0.001). As well, the number of children and family income predicted the risk of household FI. FI was around 7 times higher among households having more than one child (vs. one child; OR = 6.7, CI = 3.0–14.9, *p* < 0.001). Besides, households having no or less than 1.5 million LBP monthly income were 2.4 times more likely to be food-insecure (OR = 2.4, CI = 1.4–4.3, *p* = 0.004) (Model 1; Table 4).

**TABLE 4:** The contribution of the adolescents’ NL and parental FL to predicting the household and adolescents’ self-reported FI and adolescents’ nutrition status.

Model 1: The dependent variable is the household food security (food-secure (reference) vs. food-insecure)	OR (95 % CI)	*P*-value
**Parental FL (Reference: Adequate)**		
Poor	2.7 (1.8–4.3)	<0.001**
Number of children (Reference: One child)	1.0	–
More than one child	6.7 (3.0–14.9)	<0.001**
Monthly income of the family (Reference: > 1.5 million LBP)		
None or less than 1.5 million LBP	2.4 (1.4–4.3)	0.004*
**Model 2: The dependent variable is the adolescents’ self-reported food security (food-secure (reference) vs. food-insecure)**		
Adolescent’s NL (Reference: Adequate)	1.0	–
Poor	1.6 (1.1–2.3)	0.02*
Residence (Reference: Other governorates)		
Akkar	4.5 (1.8–11.1)	0.001*
Household crowding (Reference: No)		
Yes	1.4 (1.0–2.2)	0.04*

OR, odds ratio; CI, confidence interval; *significant at *p*-value < 0.05; **significant at *p*-value of <0.001.

Nutritionally illiterate adolescents were 60% more likely to self-experience FI (OR = 1.6, CI = 1.1–2.3, *p* = 0.02). In addition, adolescents residing in Akkar (OR = 4.5, CI = 1.8–11.1, *p* < 0.001), and in crowded households (OR = 1.6, CI = 1.1–2.3, *p* = 0.02) were 4.5 and 1.4 times more probable to be food-insecure, respectively (Model 2; Table 4).

## Discussion

This is the first study of its kind in Lebanon and the Arab region that investigated the impact of NFL among parent’s-adolescents dyads on household’s food insecurity, malnutrition and anemia.

In the MENA region, the studies addressing NL and FL status are scant so far. This is evident in a recent review (27) on the FL and NL status, which warns regional researchers to start taking incremental steps to address these concepts in the MENA region. In Lebanon, only one preliminary study was conducted to assess the NL of adolescents and observed a lower percentage of poor NL (21.2%) than that currently reported (28). Moreover, our findings with regards to adolescents’ NL is similar to that reported among Chinese (27), and Turkish adolescents (29, 30). Our findings are warrantable and could be partially explained by the fact that almost all (91.0%) of the adolescents reported not receiving nutrition education in their schools’ curriculum in the current study. Schools are ideal systems that provide a unique platform to enhance nutrition awareness by providing a variety of interventions and extracurricular activities (31). In this regard, the school-based program Teens Eating for Energy and Nutrition at School (TEENS) was a success in encouraging adolescents to choose lower-fat food selections from the cafeteria (32). Besides, a nutrition education intervention, delivered for 3 months in schools, improved adolescents’ knowledge, attitude, and behaviors related to breakfast in Indonesia (33). These results are therefore encouraging to be taken into consideration at the Lebanese schools, which of course calls for a multisector discussion between the Ministry of Education and Higher Education (MEHE), the Ministry of Public Health (MOPH), and, of course, the recently established Lebanese Order of Dietitians. School-based interventions to improve NL are many and include classroom activities, farm-to-school programs, and school garden programs (31). The advantage of nutrition education might be extended to include other children at home as well as the guardians through morning text messages with nutrition-related material, assigning cooking tasks, and group interviews with parents (31). The present findings also reveal that boys were more nutritionally illiterate than girls, *p* = 0.002. Girls are usually concerned about the energy and nutrition content of the food they eat due to body image concerns and body weight idealism objectives, particularly in adolescence (28). Furthermore, nearly half of the parents were food-illiterate in the current study. This finding is supportive of previous research emphasizing the need to develop parental FL programs to promote healthy eating habits for their families (34). In Greece, higher parental levels of FL predicted adequate feeding practices for their children (35). To attain nutrition goals, it is therefore insufficient to merely target adolescents through school-based or other initiatives; instead, it is essential to include parents in large-scale efforts.

As outlined in our findings, poor NL among adolescents and inadequate FL among their parents could lead adolescents to have an additional burden of malnutrition prevalence. Stunting, wasting, overweight, obesity, and anemia were all considerably prevalent among our adolescent sample and comparable to other studies (35–37). This is, of course, a finding to be worried about, since Lebanese adolescents are anticipated to face additional risks of malnutrition, due to the ongoing conflicts they are already dealing with. As a result, a lack of NL will cause the problem to escalate to terrifying levels.

### FL and NL programs relieve FI in poor and wealthy communities

In the current study, poor parental FL and adolescent NL had predicted worse food security status of the sampled households and the adolescents, respectively. These findings supported research from Australia (10) and Iran (13). Similarly, lower cooking self-efficacy and food preparation skills were observed among food-insecure college students (37–39). Today, policymakers and nutrition researchers embrace FL programs as a tool to minimize FI because they emphasize the utilization pillar of food security (10). Nonetheless, the relationship between FL and FI is dual (10). What if I possess adequate FL; however, the food is unavailable or ridiculously expensive? FI might still exist. Likewise, FL is not an exemplar that will eradicate FI under all circumstances. Many immutable factors contribute to causing FI, including the monthly income of the family, limited access to food due to high prices or poor infrastructure, residency, and education (10). These are evidenced in the current study, as the monthly income of the family and the number of children the parent had determined the risk of household FI, along with the parental FL status. In addition, adolescents’ residence and the crowding status of the households determined the risk of adolescents’ self-experienced FI. Ultimately, FL is one of the countless risk factors that shape FI in a population. However, it is crucial to focus on it because it is highly malleable and amenable to interventions.

### Food and nutrition literacy interventions could help build resilience against FI in lebanon

FI affects more than half the Lebanese population, according to much evidence (14–16, 40–42). The current study showed worse food security status in Lebanon among parents-adolescents dyads. Joblessness, stagnating income, declining purchasing power due to insane inflation, and a lack of social safety nets have had a significant negative impact on livelihoods (43). There is little chance of escaping or finding relief from the current economic crisis in Lebanon, thanks to the poor governance and the “no” actions taken to strengthen the national food system. Thus, FL and NL interventions could help in building resilience against FI in Lebanon. Schools, without a doubt, could be the perfect place to get in touch with adolescents and their parents and begin interfering with their food and nutrition literacy levels.

## Study limitations and strengths

There are some study limitations that the authors have to acknowledge. This is a cross-sectional study; therefore, associations between study variables could be driven with no causality. Due to the unavailability of valid nutrition and food literacy questionnaires in Lebanon, we used the Arabic translated questionnaires from Swiss and Turkish studies. However, this study is the first of its kind enrolling a representative sample of parent-adolescent dyads with the aforementioned objectives.

## Conclusion and implications

A significant proportion of Lebanese adolescents were nutrition illiterate, and nearly half of their parents had poor FL levels. Inadequate levels of adolescents’ NL and parental FL worsened adolescents’ and households’ food security status, respectively. Today, amid the national overwhelming crises, FL and NL interventions should be considered in Lebanon, preferably in school settings, to mitigate the devastating effects of FI and malnutrition. Investments in education sectors are promising to lead to durable, sustainable and effective nutrition outcomes; however, this calls for agility, resiliency, and tenacity from all stakeholders.

## Data availability statement

The original contributions presented in this study are included in the article/Supplementary material, further inquiries can be directed to the corresponding author.

## Ethics statement

The studies involving human participants were reviewed and approved by Al Zahraa University Medical Center Beirut Lebanon. Written informed consent to participate in this study was provided by the participants or their legal guardian/next of kin.

## Author contributions

MH and HM contributed to the conceptualization, data curation, formal analysis, investigation, methodology, project administration, and writing—original draft preparation. YS and LH-W contributed to the writing—review and editing. MH was responsible for supervision and validation. All authors have read and agreed to the published version of the manuscript.

## References

[1] Sustain Ontario. *Food Literacy Student Nutrition Programs & Food Literacy.* (2013). Available online at: https://sustainontario.com/greenhouse/custom/uploads/2016/09/Food-literacy-and-SNPs.pdf (accessed 14 August, 2022).

[2] CDC. *Food Literacy, Nutritional Literacy & Health Literacy.* (2022). Available online at: https://www.cdc.gov/healthliteracy/researchevaluate/foodliteracy.html#:~:text=Children%20and%20food%20literacy (accessed 14 August, 2022).

[3] Liu T Su X Li N Sun J Ma G Zhu W. Development and validation of a food and Nutrition Literacy questionnaire for Chinese school-age children. *PLoS One.* (2021) 16:e0244197. 10.1371/journal.pone.0244197 33406105 PMC7787443

[4] Lee C-K Liao L-L Lai I-J Chang L-C. Effects of a healthy-eater self-schema and Nutrition Literacy on healthy-eating behaviors among Taiwanese college students. *Health Promot Int.* (2019) 34:269–76. 10.1093/heapro/dax080 29149269

[5] Ronto R Ball L Pendergast D Harris N. Adolescents’ perspectives on Food Literacy and its impact on their dietary behaviours. *Appetite.* (2016) 107:549–57. 10.1016/j.appet.2016.09.006 27614212

[6] Kahssay M Mohamed L Gebre A. Nutritional status of school going adolescent girls in awash town, afar region, Ethiopia. *J Environ Public Health.* (2020) 2020:7367139. 10.1155/2020/7367139 32148529 PMC7054789

[7] Ashoori M Omidvar N Eini-Zinab H Shakibazadeh E Doustmohamadian A Abdar-Esfahani B Food and nutrition literacy status and its correlates in iranian senior high-school students. *BMC Nutr.* (2021) 7:19. 10.1186/s40795-021-00426-2 34082827 PMC8176697

[8] Doustmohammadian A Omidvar N Keshavarz-Mohammadi N Eini-Zinab H Amini M Abdollahi M Low food and Nutrition Literacy (FNLIT): A barrier to dietary diversity and nutrient adequacy in school age children. *BMC Res Notes.* (2020) 13:286. 10.1186/s13104-020-05123-0 32532341 PMC7291429

[9] Li S Zhu Y Zeng M Li Z Zeng H Shi Z Association between Nutrition Literacy and overweight/obesity of adolescents: A cross-sectional study in Chongqing, China. *Front Nutr.* (2022) 9:893267. 10.3389/fnut.2022.893267 35634378 PMC9134066

[10] Begley A Paynter E Butcher L Dhaliwal S. Examining the association between food literacy and food insecurity. *Nutrients.* (2019) 11:445. 10.3390/nu11020445 30791670 PMC6412525

[11] USDA. *Definitions of food security.* (2022). Available online at: https://www.ers.usda.gov/topics/food-nutrition-assistance/food-security-in-the-u-s/definitions-of-food-security/ (accessed 20 August, 2022).

[12] West E Lindberg R Ball K McNaughton S. The role of a Food Literacy intervention in promoting food security and Food Literacy-OzHarvest’s NEST program. *Nutrients.* (2020) 12:2197. 10.3390/nu12082197 32718054 PMC7468773

[13] Khorramrouz F Doustmohammadian A Eslami O Khadem-Rezaiyan M Pourmohammadi P Amini M Relationship between household Food Insecurity and food and Nutrition Literacy among children of 9-12 years of age: a cross-sectional study in a city of Iran. *BMC Res Notes.* (2020) 13:433. 10.1186/s13104-020-05280-2 32933579 PMC7493354

[14] Hoteit M Al-Atat Y Joumaa H Ghali SE Mansour R Mhanna R Exploring the impact of crises on food security in lebanon: Results from a National Cross-Sectional Study. *Sustainability.* (2021) 13:8753. 10.3390/su13168753

[15] Hoteit M Mortada H Al-Jawaldeh A Ibrahim C Mansour R. COVID-19 home isolation and food consumption patterns: Investigating the correlates of poor dietary diversity in Lebanon: a cross-sectional study. *F1000Research.* (2022) 11:110. 10.12688/f1000research.75761.1 35251599 PMC8864186

[16] Yazbeck N Mansour R Salame H Chahine NB Hoteit M. The ukraine-russia war is deepening food insecurity, unhealthy dietary patterns and the lack of dietary diversity in lebanon: Prevalence, correlates and findings from a national cross-sectional study. *Nutrients.* (2022) 14:3504. 10.3390/nu14173504 36079761 PMC9460330

[17] Lemeshow S. Sample size determination in health studies. In: Lwanga SK editor. *A Practical Manual.* Geneva: World Health Organization (1991).

[18] Central Administration of Statistics, (2022). Available online at: http://www.cas.gov.lb/ (accessed February 12, 2022).

[19] Hoteit M. *Food Literacy Study Questionnaires.* Peoria, IL: OSF (2022). 10.17605/OSF.IO/Q94JE

[20] Türkmen A Kalkan I Filiz E. Adaptation of adolescent nutrition literacy scale into Turkish: A validity and reliability study. *Int Peer-Rev J Nutr Res.* (2017) 10:1–16. 10.17362/dbhad.2017.2.01

[21] Krause C Sommerhalder K Beer-Borst S Abel T. Just a subtle difference? Findings from a systematic review on definitions of nutrition literacy and food literacy. *Health Promot Int.* (2016) 33:daw084. 10.1093/heapro/daw084 27803197 PMC6005107

[22] Gréa Krause C Beer-Borst S Sommerhalder K Hayoz S Abel T. A short food literacy questionnaire (SFLQ) for adults: Findings from a Swiss validation study. *Appetite.* (2018) 120:275–80. 10.1016/j.appet.2017.08.039 28912107

[23] Sahyoun N Nord M Sassine A Seyfert K Hwalla N Ghattas H. Development and validation of an Arab family food security scale. *J Nutr.* (2014) 144:751–7. 10.3945/jn.113.187112 24598883

[24] Jamaluddine Z Sahyoun N Choufani J Sassine A Ghattas H. Child-reported food insecurity is negatively associated with household food security, socioeconomic status, diet diversity, and school performance among children attending UN Relief and Works Agency for Palestine refugee’s schools in Lebanon. *J Nutr.* (2019) 149:2228–35. 10.1093/jn/nxz189 31504697

[25] World Health Organization. *WHO Growth Reference.* Geneva: World Health Organization (2007).

[26] World Health Organization. *Hemoglobin concentrations for the diagnosis of anemia and assessment of severity.* (2011). Available online at: https://apps.who.int/iris/handle/10665/85839. (accessed 26 July, 2022).

[27] Mohsen H Sacre Y Hanna-Wakim L Hoteit M. Nutrition and food literacy in the MENA region: A review to inform nutrition research and policy makers. *Int J Environ Res Public Health.* (2022) 19:10190. 10.3390/ijerph191610190 36011837 PMC9408592

[28] Taleb S Itani L. Nutrition literacy among adolescents and its association with eating habits and BMI in Tripoli, Lebanon. *Diseases.* (2021) 9:25. 10.3390/diseases9020025 33805571 PMC8103266

[29] Zeng M Zhu Y Cai Z Xian J Li S Wang T Nutrition literacy of middle school students and its influencing factors: A cross-sectional study in Chongqing, China. *Front Public Health.* (2022) 10:807526. 10.3389/fpubh.2022.807526 35372191 PMC8965039

[30] Ayer Ç Ergin A. Status of nutritional literacy in adolescents in the semi-rural area in Turkey and related factors. *Public Health Nutr.* (2021) 24:3870–8. 10.1017/S1368980021002366 34047263 PMC8369453

[31] Koca B Arkan G. The relationship between adolescents’ nutrition literacy and food habits, and affecting factors. *Public Health Nutr.* (2020) 24:1–12. 10.1017/S1368980020001494 32723409 PMC11574834

[32] United Nations System Standing Committee on Nutrition (UNSCN). *Schools as a System to Improve Nutrition A new statement for school-based food and nutrition interventions.* (2017). Available online at: https://www.unscn.org/uploads/web/news/document/School-Paper-EN-WEB-nov2017.pdf. (accessed August 15, 2022).

[33] E C Control Programs (EBCCP). *Teens Eating for Energy and Nutrition at School (TEENS).* (2020). Available online at: https://ebccp.cancercontrol.cancer.gov/programDetails.do?programId=246210 (accessed June 14, 2022).

[34] Indriasari R Nadjamuddin U Arsyad D Iswarawanti D. School-based nutrition education improves breakfast-related personal influences and behavior of Indonesian adolescents: a cluster randomized controlled study. *Nutr Res Pract.* (2021) 15:639–54. 10.4162/nrp.2021.15.5.639 34603611 PMC8446687

[35] Tartaglia J McIntosh M Jancey J Scott J Begley A. Exploring feeding practices and food literacy in parents with young children from disadvantaged areas. *Int J Environ Res Public Health.* (2021) 18:1496. 10.3390/ijerph18041496 33557440 PMC7915516

[36] Costarelli V Michou M Panagiotakos D Lionis C. Parental health literacy and nutrition literacy affect child feeding practices: A cross-sectional study. *Nutr Health.* (2022) 28:59–68. 10.1177/02601060211001489 33913343

[37] Tarini N Sugandini W Sulyastini N. Prevalence of anemia and stunting in early adolescent girls. In *Proceedings of the 3rd International Conference on Innovative Research Across Disciplines.* Paris: Atlantis Press (2019). 2020 p. 10.2991/assehr.k.200115.065

[38] Talat M El Shahat E. Prevalence of overweight and obesity among preparatory school adolescents in Urban Sharkia Governorate, Egypt. *Egypt Pediatr Assoc Gaz.* (2016) 64:20–5.

[39] Shaban L Al-Taiar A Rahman A Al-Sabah R Mojiminiyi O. Anemia and its associated factors among Adolescents in Kuwait. *Sci Rep.* (2020) 10:5857. 10.1038/s41598-020-60816-7 32246050 PMC7125127

[40] Knol L Robb C McKinley E Wood M. Very low food security status is related to lower cooking self-efficacy and less frequent food preparation behaviors among college students. *J Nutr Educ Behav.* (2019) 51:357–63. 10.1016/j.jneb.2018.10.009 30528982

[41] Jomaa L Naja F Cheaib R Hwalla N. Household food insecurity is associated with a higher burden of obesity and risk of dietary inadequacies among mothers in Beirut, Lebanon. *BMC Public Health.* (2017) 17:567. 10.1186/s12889-017-4317-5 28606120 PMC5469040

[42] Jomaa L Naja F Kharroubi S Hwalla N. Prevalence and correlates of Food Insecurity among Lebanese households with children aged 4–18 years: Findings from a national cross-sectional study. *Public Health Nutr.* (2019) 22:202–11. 10.1017/S1368980018003245 30511613 PMC6390393

[43] World Food Program. *Lebanon.* (2022). Available online at: https://www.wfp.org/countries/lebanon (accessed 15 August, 2022).

