# Supplementary material for: Parent’s food literacy and adolescents nutrition literacy influence household’s food security and adolescent’s malnutrition and anemia: Findings from a national representative cross sectional study
Source: Front Nutr. 2022 Dec 30;9:1053552. doi: 10.3389/fnut.2022.1053552 (PMC9837989; doi:10.3389/fnut.2022.1053552)
Supplement: Supplementary file 1 [file Table_1.docx]

**Table S1.** The Relationships Between Parental and Adolescent characteristics, and the Adolescents' NL and Parental FL, with the Household and Self-Reported Adolescent Food Security and Adolescents' Nutrition Status.

|  | **Household food security** | | **p-value ^(a)^** | **Adolescents’ self-reported food security** | | **p-value**  **^(b)^** | **Stunting** | | **p-value**  **^(c)^** | **Thinness** | | **p-value**  **^(d)^** | **Overweight/ Obesity** | | **p-value**  **^(e)^** | **Anemia** | | **p-value ^(f)^** |
| --- | --- | --- | --- | --- | --- | --- | --- | --- | --- | --- | --- | --- | --- | --- | --- | --- | --- | --- |
|  | **Food-secure** | **Food-insecure** |  | **Food-secure** | **Food-insecure** |  | **No** | **Yes** |  | **No** | **Yes** |  | **No** | **Yes** |  | **No** | **Yes** |  |
|  | n (%) | n (%) |  | n (%) | n (%) |  | n (%) | n (%) |  | n (%) | n (%) |  | n (%) | n (%) |  | n (%) | n (%) |  |
| **Adolescents’ gender** |  |  | 0.16 |  |  | 0.59 |  |  | 0.54 |  |  | 0.004* |  |  | 0.09 |  |  | 0.80 |
| Girl | 85  (34.6) | 161  (65.4) |  | 116  (47.2) | 13  (52.8) |  | 228 (92.7) | 18 (7.3) |  | 241 (98.0) | 5 (2.0) |  | 175 (71.1) | 71 (28.9) |  | 206 (83.7) | 40 (16.3) |  |
| Boy | 58  (28.4) | 146  (71.6) |  | 91  (44.6) | 113  (55.4) |  | 192 (94.1) | 12 (5.9) |  | 188 (92.2) | 16 (7.8) |  | 130 (63.7) | 74 (36.3) |  | 169 (82.8) | 35 (17.2) |  |
| **Adolescents’ age** |  |  | 0.31 |  |  | 0.65 |  |  | 0.13 |  |  | 0.48 |  |  | 0.70 |  |  | 0.85 |
| Early adolescence | 52  (28.9) | 128  (71.1) |  | 78  (43.3) | 102  (56.7) |  | 164 (91.1) | 16 (8.9) |  | 169 (93.9) | 11 (6.1) |  | 124 (68.9) | 56 (31.1) |  | 152 (84.4) | 28 (15.6) |  |
| Middle adolescence | 37  (30.3) | 85  (69.7) |  | 58  (47.5) | 64  (52.5) |  | 113 (92.6) | 9  (7.4) |  | 117 (95.9) | 5  (4.1) |  | 79 (64.8) | 43 (35.2) |  | 100 (82.0) | 22 (18.0) |  |
| Late adolescence | 54  (36.5) | 94  (63.5) |  | 71  (48.0) | 77  (52.0) |  | 143 (96.6) | 5  (3.4) |  | 143 (96.6) | 5  (3.4) |  | 102 (68.9) | 46 (31.1) |  | 123 (83.1) | 25 (16.9) |  |
| **Area of residency** |  |  | <0.001** |  |  | <0.001** |  |  | 0.58 |  |  | 0.75 |  |  | 0.12 |  |  | 0.01* |
| Mount Lebanon | 54  (32.1) | 114  (67.9) |  | 90  (53.6) | 78  (46.4) |  | 153 (91.1) | 15 (8.9) |  | 158 (94.0) | 10 (6.0) |  | 108 (64.3) | 60 (35.7) |  | 144 (85.7) | 24 (14.3) |  |
| Beirut | 8  (30.8) | 18  (69.2) |  | 10  (38.5) | 16  (61.5) |  | 26 (100.0) | 0  (0.0) |  | 25  (96.2) | 1  (3.8) |  | 20 (76.9) | 6 (23.1) |  | 22  (84.6) | 4 (15.4) |  |
| South Lebanon | 34  (54.0) | 29 (46.0) |  | 40  (63.5) | 23  (36.5) |  | 60 (95.2) | 3  (4.8) |  | 60  (95.2) | 3  (4.8) |  | 47 (74.6) | 16 (25.4) |  | 57  (90.5) | 6  (9.5) |  |
| North Lebanon | 22  (37.9) | 36  (62.1) |  | 17  (29.3) | 41  (70.7) |  | 56 (96.6) | 2  (3.4) |  | 57  (98.3) | 1  (1.7) |  | 40 (69.0) | 18 (31.0) |  | 43  (74.1) | 15 (25.9) |  |
| Akkar | 7  (17.9) | 32  (82.1) |  | 6  (15.4) | 33  (84.6) |  | 36 (92.3) | 3  (7.7) |  | 37  (94.9) | 2  (5.1) |  | 23 (59.0) | 16 (41.0) |  | 25  (64.1) | 14 (35.9) |  |
| Beqaa | 6  (19.4) | 25  (80.6) |  | 12  (38.7) | 19  (61.3) |  | 28 (90.3) | 3  (9.7) |  | 31 (100.0) | 0  (0.0) |  | 27 (87.1) | 4 (12.9) |  | 27  (87.1) | 4 (12.9) |  |
| Baalbeck-Hermel | 5  (17.9) | 23  (82.2) |  | 10  (35.7) | 18  (64.3) |  | 27 (96.4) | 1  (3.6) |  | 27  (96.4) | 1  (3.6) |  | 18 (64.3) | 10 (35.7) |  | 25  (89.3) | 3 (10.7) |  |
| Nabatieh | 7  (18.9) | 30  (81.1) |  | 22  (59.5) | 15  (40.5) |  | 34 (91.9) | 3  (8.1) |  | 34  (91.9) | 3  (8.1) |  | 22 (59.5) | 15 (40.5) |  | 32  (86.5) | 5 (13.5) |  |
| **Adolescents’ education level** |  |  | 0.95 |  |  | 0.87 |  |  | 0.02* |  |  | 0.27 |  |  | 0.09 |  |  | 0.92 |
| School education level | 114  (31.8) | 244  (68.2) |  | 164  (45.8) | 194  (54.2) |  | 329 (91.9) | 29 (8.1) |  | 339 (94.7) | 19 (5.3) |  | 236 (65.9) | 122 (34.1) |  | 298 (83.2) | 60 (16.8) |  |
| University education level | 29  (31.5) | 63  (68.5) |  | 43  (46.7) | 49  (53.3) |  | 91 (98.9) | 1  (1.1) |  | 90  (97.8) | 2 (2.2) |  | 69 (75.0) | 23 (25.0) |  | 77  (83.7) | 15 (16.3) |  |
| **The adolescent is receiving nutrition education at school** |  |  | 0.55 |  |  | 0.13 |  |  | 0.65 |  |  | 0.15 |  |  | 0.49 |  |  | 0.45 |
| No | 105  (32.3) | 220  (67.7) |  | 153  (47.1) | 172  (52.9) |  | 298  (91.7) | 27  (8.3) |  | 306  (94.2) | 19  (5.8) |  | 216  (66.5) | 109  (33.5) |  | 269  (82.8) | 56  (17.2) |  |
| **Yes** | 9  (27.3) | 24  (72.7) |  | 11  (33.3) | 22  (66.7) |  | 31  (93.9) | 2  (6.1) |  | 33  (100.0) | 0  (0.0) |  | 20  (60.6) | 13  (39.4) |  | 29  (87.9) | 4  (12.1) |  |
| **Primary caregiver** |  |  | 0.26 |  |  | 0.35 |  |  | 0.34 |  |  | 0.51 |  |  | 0.15 |  |  | 0.23 |
| Either parent | 7  (41.2) | 10  (58.8) |  | 5  (29.4) | 12  (70.6) |  | 17  (100.0) | 0  (0.0) |  | 17  (100.0) | 0  (0.0) |  | 10  (58.8) | 7  (41.2) |  | 15  (88.2) | 2  (11.8) |  |
| Both (mother and father) | 130  (30.9) | 291  (69.1) |  | 197  (46.8) | 224  (53.2) |  | 391  (92.9) | 30  (7.1) |  | 400 (95.0) | 21 (5.0) |  | 284 (67.5) | 137 (32.5) |  | 348 (82.7) | 73 (17.3) |  |
| Other caregivers | 6  (50.0) | 6  (50.0) |  | 5 (41.7) | 7  (58.3) |  | 12  (100.0) | 0  (0.0) |  | 12 (100.0) | 0  (0.0) |  | 11 (91.7) | 1  (8.3) |  | 12 (100.0) | 0  (0.0) |  |
| **Adolescents’ working status** |  |  | 0.05 |  |  | 0.08 |  |  | **0.25** |  |  | **0.65** |  |  | 0.42 |  |  | 0.11 |
| No | 138  (32.9) | 281  (67.1) |  | 188  (44.9) | 231  (55.1) |  | 393 (93.8) | 26 (6.2) |  | 400 (95.5) | 19 (4.5) |  | 286 (68.3) | 133 (31.7) |  | 346 (82.6) | 73 (17.3) |  |
| Yes | 5  (16.1) | 26  (83.9) |  | 19  (61.3) | 12  (38.7) |  | 27 (87.1) | 4 (12.9) |  | 29  (93.5) | 2 (6.5) |  | 19 (61.3) | 12 (38.7) |  | 29  (93.5) | 2  (6.5) |  |
| **Parental gender** |  |  | 0.35 |  |  | 0.20 |  |  | 0.92 |  |  | 0.52 |  |  | 0.88 |  |  | 0.86 |
| Female | 80  (30.1) | 186  (69.9) |  | 129  (48.5) | 137  (51.5) |  | 248 (93.2) | 18 (6.8) |  | 255 (95.9) | 11 (4.1) |  | 181 (68.0) | 85 (32.0) |  | 221 (83.1) | 45 (16.9) |  |
| Male | 63  (34.2) | 121  (65.8) |  | 78  (42.4) | 106  (57.6) |  | 172 (93.5) | 12 (6.5) |  | 174 (94.6) | 10 (5.4) |  | 124 (67.4) | 60 (32.6) |  | 154 (83.7) | 30 (16.3) |  |
| **Parental age** |  |  | 0.10 |  |  | 0.14 |  |  | 0.90 |  |  | 0.09 |  |  | 0.82 |  |  | 0.24 |
| Early middle adulthood | 65  (28.3) | 165  (71.7) |  | 98  (42.6) | 132  (57.4) |  | 215 (93.5) | 15 (6.5) |  | 223 (97.0) | 7  (3.0) |  | 157 (68.3) | 73 (31.7) |  | 187 (81.3) | 43 (18.7) |  |
| Late middle adulthood | 78  (35.5) | 142  (64.5) |  | 109  (49.5) | 111  (50.5) |  | 205 (93.2) | 15 (6.8) |  | 206 (93.6) | 14 (6.4) |  | 148 (67.3) | 72 (32.7) |  | 188 (85.5) | 32 (14.5) |  |
| **Parental weight status** |  |  | 0.36 |  |  | 0.49 |  |  | 0.82 |  |  | 0.31 |  |  | 0.002* |  |  | 0.14 |
| Underweight | 1  (12.5) | 7  (87.5) |  | 4  (50.0) | 4  (50.0) |  | 8 (100.0) | 0  (0.0) |  | 7  (87.5) | 1 (12.5) |  | 7 (87.5) | 1 (12.5) |  | 8  (100.0) | 0  (0.0) |  |
| Normal | 74  (33.9) | 144  (66.1) |  | 94  (43.1) | 124  (56.9) |  | 204 (93.6) | 14 (6.4) |  | 210 (96.3) | 8 (3.7) |  | 163 (74.8) | 55 (25.2) |  | 175 (80.3) | 43 (19.7) |  |
| Overweight/ Obese | 68  (30.4) | 156  (69.6) |  | 109  (48.7) | 115  (51.3) |  | 208 (92.9) | 16 (7.1) |  | 212 (94.6) | 12 (5.4) |  | 135 (60.3) | 89 (39.7) |  | 192 (85.7) | 32 (14.3) |  |
| **Parental marital status** |  |  | 0.50 |  |  | 0.77 |  |  | 0.68 |  |  | 1.0 |  |  | 0.21 |  |  | 0.26 |
| Married | 135  (31.3) | 296  (68.7) |  | 199  (46.2) | 232  (53.8) | 0.72 | 402 (93.3) | 29 (6.7) |  | 410 (95.1) | 21 (4.9) |  | 293 (68.0) | 138 (32.0) |  | 357 (82.8) | 74 (17.2) |  |
| Divorced | 3  (33.3) | 6  (66.7) |  | 3  (33.3) | 6  (66.7) |  | 8 (88.9) | 1 (11.1) |  | 9  (100.0) | 0 (0.0) |  | 4 (44.4) | 5 (55.6) |  | 8  (88.9) | 1 (11.1) |  |
| Widowed | 5  (50.0) | 5  (50.0) |  | 5  (50.0) | 5  (50.0) |  | 10 (100.0) | 0  (0.0) |  | 10 (100.0) | 0 (0.0) |  | 8 (80.0) | 2 (20.0) | 0.10 | 10 (100.0) | 0  (0.0) |  |
| **Number of children the parent has** |  |  | <0.001** |  |  | 0.07 |  |  | 0.17 |  |  | 0.09 |  |  | 0.09 |  |  | 0.58 |
| One child | 21  (61.8) | 13  (38.2) |  | 13  (38.2) | 21  (61.8) |  | 34 (100.0) | 0  (0.0) |  | 34 (100.0) | 0 (0.0) |  | 19 (55.9) | 15 (44.1) |  | 30  (88.2) | 4 (11.8) |  |
| 2-3 children | 82  (33.7) | 161  (66.3) |  | 124  (51.0) | 119  (49.0) |  | 223 (91.8) | 20 (8.2) |  | 227 (93.4) | 16 (6.6) |  | 174 (71.6) | 69 (28.4) |  | 204 (84.0) | 39 (16.0) |  |
| More than 3 children | 40  (23.1) | 133  (76.9) |  | 70  (40.5) | 103  (59.5) |  | 163 (94.2) | 10 (5.8) |  | 168 (97.1) | 5 (2.9) |  | 112 (64.7) | 61 (35.3) |  | 141 (81.5) | 32 (18.5) |  |
| **Parental education level** |  |  | 0.005* |  |  | 0.28 |  |  | 0.83 |  |  | 0.50 |  |  | 0.009* |  |  | 0.66 |
| Nor formal education | 4  (11.4) | 31  (88.6) |  | 12  (34.3) | 23  (65.7) |  | 33 (94.3) | 2  (5.7) |  | 34  (97.1) | 1 (2.9) |  | 30 (85.7) | 5 (14.3) |  | 31 (88.6) | 4 (11.4) |  |
| School level | 90  (30.8) | 202  (69.2) |  | 134  (45.9) | 158  (54.1) |  | 271 (92.8) | 21 (7.2) |  | 280 (95.9) | 12 (4.1) |  | 185 (63.4) | 107 (36.6) |  | 243 (83.2) | 49 (16.8) |  |
| University level | 49  (39.8) | 74  (60.2) |  | 61  (49.6) | 62  (50.4) |  | 116 (94.3) | 7  (5.7) |  | 115 (93.5) | 8 (6.5) |  | 90 (73.2) | 33 (26.8) |  | 101 (82.1) | 22 (17.9) |  |
| **Parental occupation** |  |  | 0.08 |  |  | 0.98 |  |  | 0.65 |  |  | 0.87 |  |  | 0.76 |  |  | 0.21 |
| No | 62  (27.9) | 160  (72.1) |  | 102  (45.9) | 120  (54.1) |  | 206 (92.8) | 16 (7.2) |  | 212 (95.5) | 10 (4.5) |  | 152 (68.5) | 70 (31.5) |  | 180 (81.1) | 42 (18.9) |  |
| Yes | 81  (35.5) | 147  (64.5) |  | 105  (46.1) | 123  (53.9) |  | 214 (93.9) | 14 (6.1) |  | 217 (95.2) | 11 (4.8) |  | 153 (67.1) | 75 (32.9) |  | 195 (85.5) | 33 (14.5) |  |
| **Monthly income of the family** |  |  | 0.01* |  |  | 0.04* |  |  | 0.58 |  |  | 0.14 |  |  | 0.78 |  |  | 0.79 |
| None or less than 1.5 million L.B.P. | 22  (21.6) | 80  (78.4) |  | 38  (37.3) | 64  (62.7) |  | 94  (92.2) | 22  (6.3) |  | 100  (98.0) | 2  (2.0) |  | 68  (66.7) | 34  (33.3) |  | 87  (85.3) | 15  (14.7) |  |
| More than 1.5 million L.B.P. | 121  (34.8) | 227  (65.2) |  | 169  (48.6) | 179  (51.4) |  | 326  (93.7) | 8  (7.8) |  | 329  (94.5) | 19  (5.5) |  | 237  (68.1) | 111  (31.9) |  | 288  (82.8) | 60  (17.2) |  |
| **The household has experienced a recent decline in the monthly income** |  |  | 0.59 |  |  | 0.24 |  |  | 0.51 |  |  | 0.02* |  |  | 0.92 |  |  | 0.92 |
| No | 114  (32.4) | 238  (67.6) |  | 167  (47.4) | 185  (52.6) |  | 330  (93.8) | 22  (6.3) |  | 340  (96.6) | 12  (3.4) |  | 239  (67.9) | 113  (32.1) |  | 291  (82.7) | 61  (17.3) |  |
| Yes | 29  (29.6) | 69  (70.4) |  | 40  (40.8) | 58  (59.2) |  | 90 (91.8) | 8  (8.2) |  | 89  (90.8) | 9  (9.2) |  | 66 (67.3) | 21 (32.7) |  | 84  (85.7) | 14 (14.3) |  |
| **Household crowding status** |  |  | 0.04* |  |  | 0.01* |  |  | 0.56 |  |  | 0.85 |  |  | 0.85 |  |  | 0.87 |
| Not crowded | 89  (35.9) | 159  (64.1) |  | 127  (51.2) | 121  (48.8) |  | 233 (94.0) | 15 (6.0) |  | 236 (95.2) | 12 (4.8) |  | 169 (68.1) | 79 (31.9) |  | 206 (83.1) | 42 (16.9) |  |
| Crowded | 54  (26.7) | 148  (73.3) |  | 80  (39.6) | 122  (60.4) |  | 187 (92.6) | 15 (7.4) |  | 193 (95.5) | 9 (4.5) |  | 136 (67.3) | 66 (32.7) |  | 169 (83.7) | 33 (16.3) |  |
| **Adolescent’s NL** |  |  | 0.11 |  |  | 0.01* |  |  | 0.41 |  |  | 0.78 |  |  | 0.80 |  |  | 0.16 |
| Poor | 56  (27.9) | 145  (72.1) |  | 79  (39.3) | 122  (60.7) |  | 185  (92.0) | 16  (8.0) |  | 191  (95.0) | 10  (5.0) |  | 135  (67.2) | 66  (32.8) |  | 162  (80.6) | 39  (19.4) |  |
| Adequate | 87  (34.9) | 162  (65.1) |  | 128  (51.4) | 121  (48.6) |  | 235  (94.4) | 14  (5.6) |  | 238  (95.6) | 11  (4.4) |  | 170  (68.3) | 79  (31.7) |  | 213  (85.5) | 36  (14.5) |  |
| **Parental FL** |  |  | <0.001** |  |  | 0.35 |  |  | 0.80 |  |  | 0.64 |  |  | 0.73 |  |  | 0.64 |
| Poor | 47  (21.9) | 168  (78.1) |  | 94  (43.7) | 121  (56.3) |  | 200  (93.0) | 15  (7.0) |  | 206  (95.8) | 9  (4.2) |  | 144  (67.0) | 71  (33.0) |  | 181  (84.2) | 34  (15.8) |  |
| Adequate | 96  (40.9) | 139  (59.1) |  | 113  (48.1) | 122  (51.9) |  | 220  (93.6) | 15  (6.4) |  | 223  (94.9) | 12  (5.1) |  | 161  (68.5) | 74  (31.5) |  | 194  (82.6) | 41  (17.4) |  |

1. p-value related to the household food security status variable.; (b) p-value related to the adolescents’ self-reported food security status variable; (c) p-value related to stunting variable; (d) p-value related to thinness variable; (e) p-value related to overweight/obesity variable.; (f) p-value related to anemia variable; *significant at p-value <0.05; ** significant at p-value of <0.001; bold values are determined based on Fisher’s exact test.
